# Supplementary material for: Group B Streptococcus Cas9 variants provide insight into programmable gene repression and CRISPR-Cas transcriptional effects
Source: Commun Biol. 2023 Jun 9;6:620. doi: 10.1038/s42003-023-04994-w (PMC10256743; doi:10.1038/s42003-023-04994-w)
Supplement: Supplementary file 3 — Description of Additional Supplementary Files [file 42003_2023_4994_MOESM3_ESM.pdf]

Description of Additional Supplementary Files

File name: Supplemental Data 1

Description: DESeq2 output for RNA-seq comparisons using A909 covR knockdowns, CNCTC 10/84 cyl knockdowns, and sham targeted controls.

File name: Supplemental Data 2

Description: DESeq2 output for RNA-seq comparisons of cas9 variants of CNCTC 10/84.

File name: Supplemental Data 3

Description: Tables with GSEA leading edge analyses for COG categories F, G, and V.

File name: Supplemental Data 4

Description: Tables with GSEA leading edge analyses for COG categories F, G, and V.

File name: Supplemental Data 5

Description: Tables with GSEA leading edge analyses for COG categories F, G, and V.

File name: Supplemental Data 6

Description: Read-count normalized expression of genes shown in the heatmap in main Figure 7E.

File name: Supplemental Data 7

Description: Numeric data for all charts.
