# Supplementary material for: Group B Streptococcus Cas9 variants provide insight into programmable gene repression and CRISPR-Cas transcriptional effects
Source: Commun Biol. 2023 Jun 9;6:620. doi: 10.1038/s42003-023-04994-w (PMC10256743; doi:10.1038/s42003-023-04994-w)
Supplement: Supplementary file 11 — Reporting Summary [file 42003_2023_4994_MOESM11_ESM.pdf]

nature portfolio

Corresponding author(s): Thomas Hooven

Last updated by author(s): 5/23/2023

Reporting Summary

Nature Portfolio wishes to improve the reproducibility of the work that we publish. This form provides structure for consistency and transparency in reporting. For further information on Nature Portfolio policies, see our Editorial Policies and the Editorial Policy Checklist.

Statistics

For all statistical analyses, confirm that the following items are present in the figure legend, table legend, main text, or Methods section.

n/a	Confirmed	
☐	☒	The exact sample size (*n*) for each experimental group/condition, given as a discrete number and unit of measurement
☐	☒	A statement on whether measurements were taken from distinct samples or whether the same sample was measured repeatedly
☐	☒	The statistical test(s) used AND whether they are one- or two-sided<br>*Only common tests should be described solely by name; describe more complex techniques in the Methods section.*
☒	☐	A description of all covariates tested
☐	☒	A description of any assumptions or corrections, such as tests of normality and adjustment for multiple comparisons
☐	☒	A full description of the statistical parameters including central tendency (e.g. means) or other basic estimates (e.g. regression coefficient) AND variation (e.g. standard deviation) or associated estimates of uncertainty (e.g. confidence intervals)
☐	☒	For null hypothesis testing, the test statistic (e.g. *F*, *t*, *r*) with confidence intervals, effect sizes, degrees of freedom and *P* value noted<br>*Give P values as exact values whenever suitable.*
☒	☐	For Bayesian analysis, information on the choice of priors and Markov chain Monte Carlo settings
☒	☐	For hierarchical and complex designs, identification of the appropriate level for tests and full reporting of outcomes
☒	☐	Estimates of effect sizes (e.g. Cohen's *d*, Pearson's *r*), indicating how they were calculated

Our web collection on statistics for biologists contains articles on many of the points above.

Software and code

Policy information about availability of computer code

Data collection	No software was used for data collection.
Data analysis	Only publicly available commercial and non-commercial software was used for data analysis. There was no development of custom software or algorithms. GraphPad Prism (MacOS version 9) was used for standard statistical testing of experimental data. Next-generation sequencing alignments were performed with Bowtie2 (version 2.4.5). Raw read alignments were quantified with HTSeq (version 2.0.2). DESeq2 (version 3.16) was used for statistical analyses of RNA-seq data. The following DeepTools (version 3.5.1) analysis algorithms were performed on a dedicated instantiation of the Galaxy bioinformatics system maintained and run by the University of Pittsburgh Center for Research Computing: multiBAMSummary, computeMatrix, plotCorrelation, and plotPCA. Upset plots were generated using the UpsetR package (version 1.4.0). GBS gene COG categories were determined with eggnog-Mapper (version 2.1.9). Gene set enrichment analyses were performed with the GSEA software package (version 4.3.2).

For manuscripts utilizing custom algorithms or software that are central to the research but not yet described in published literature, software must be made available to editors and reviewers. We strongly encourage code deposition in a community repository (e.g. GitHub). See the Nature Portfolio guidelines for submitting code & software for further information.

Data

Policy information about availability of data

All manuscripts must include a data availability statement. This statement should provide the following information, where applicable:

- Accession codes, unique identifiers, or web links for publicly available datasets
- A description of any restrictions on data availability
- For clinical datasets or third party data, please ensure that the statement adheres to our policy

Illumina whole-genome sequence and RNA-seq data are publicly available on NCBI under umbrella BioProject accession PRJNA949218 (https://www.ncbi.nlm.nih.gov/bioproject/949218). Remaining data supporting the findings of this study are available within the paper and its Supplementary Information.

Human research participants

Policy information about studies involving human research participants and Sex and Gender in Research.

Reporting on sex and gender	Human research participants were only involved in this study as volunteer donors of blood samples for GBS hemolysis assays. Neither sex nor gender is expected to affect results of these assays, nor were sex or gender self-reports collected.
Population characteristics	Phlebotomy volunteers were healthy (by self-attestation) adults over 18 years old. No further population characteristics were collected or recorded.
Recruitment	Recruitment was by direct approach. Volunteers were informed of the study purpose and the minor risks incurred by antecubital venous phlebotomy, and were offered full opportunity to decline participation. No compensation was provided.
Ethics oversight	The phlebotomy and subsequent hemolysis assay portions of this study are approved under an active University of Pittsburgh IRB protocol (19110106).

Note that full information on the approval of the study protocol must also be provided in the manuscript.

Field-specific reporting

Please select the one below that is the best fit for your research. If you are not sure, read the appropriate sections before making your selection.

☒ Life sciences ☐ Behavioural & social sciences ☐ Ecological, evolutionary & environmental sciences

For a reference copy of the document with all sections, see nature.com/documents/nr-reporting-summary-flat.pdf

Life sciences study design

All studies must disclose on these points even when the disclosure is negative.

Sample size	GBS experiment sample sizes were determined based on past experience with the assays, which showed significant differences when duplicate or triplicate technical replicates were used across two biological replicates. Mouse experiment sample sizes were based on power calculations to detect 80% differences in bacterial load with a power of 80% and alpha=0.05.
Data exclusions	No data exclusions.
Replication	All experiments were conducted with independent biological replicates as described in the manuscript.
Randomization	Not applicable.
Blinding	No blinding was performed.

Reporting for specific materials, systems and methods

We require information from authors about some types of materials, experimental systems and methods used in many studies. Here, indicate whether each material, system or method listed is relevant to your study. If you are not sure if a list item applies to your research, read the appropriate section before selecting a response.

Materials & experimental systems

n/a	Involved in the study
☒	☐ Antibodies
☒	☐ Eukaryotic cell lines
☒	☐ Palaeontology and archaeology
☐	☒ Animals and other organisms
☒	☐ Clinical data
☒	☐ Dual use research of concern

Methods

n/a	Involved in the study
☒	☐ ChIP-seq
☒	☐ Flow cytometry
☒	☐ MRI-based neuroimaging

Animals and other research organisms

Policy information about studies involving animals; ARRIVE guidelines recommended for reporting animal research, and Sex and Gender in Research

Laboratory animals	BALB/cJ mice were purchased from Jackson Laboratories. Mice were housed in specific pathogen-free conditions.
Wild animals	N/A
Reporting on sex	Only female mice were used to avoid pregnancy and sexual aggression between co-housed animals. The murine sepsis model used in this study is not known to exhibit any sex-specific differences.
Field-collected samples	N/A
Ethics oversight	All mouse experiments were performed in an American Association for the Accreditation of Laboratory Animal Care-accredited animal facility at the University of Pittsburgh and housed in accordance with the procedures outlined in the Guide for the Care and Use of Laboratory Animals under an animal study protocol (20016575) approved by the Institutional Animal Care and Use Committee of the University of Pittsburgh.

Note that full information on the approval of the study protocol must also be provided in the manuscript.
